# Transplantation of three mesenchymal stem cells for knee osteoarthritis, which cell and type are more beneficial? a systematic review and network meta-analysis

**DOI:** 10.1186/s13018-024-04846-1

**Published:** 2024-06-20

**Authors:** Xiyang Chen, Jinglu Zheng, Li Yin, Yikai Li, Hongwen Liu

**Affiliations:** 1 Zhongshan Hospital of Traditional Chinese Medicine Affiliated to Guangzhou University of Traditional Chinese Medicine, Zhongshan, Guangdong Province China; 2 Department of Discipline Construction Office, Panzhihua Central Hospital, Panzhihua, Sichuan Province China; 3 Department of Traditional Chinese Orthopedics and Traumatology, Center for Orthopaedic Surgery, The Third Affiliated Hospital of Southern Medical University, Guangzhou, Guangdong Province China; 4 School of Traditional Chinese Medicine, Southern Medical University, Guangzhou, Guangdong Province China

**Keywords:** Mesenchymal stem cells, Stem cell types, Knee, Osteoarthritis, Outcomes

## Abstract

**Background:**

In knee osteoarthritis (KOA), treatments involving knee injections of bone marrow-derived mesenchymal stem cells (BM-MSC), adipose tissue-derived mesenchymal stem cells (AD-MSC), or umbilical cord-derived mesenchymal stem cells (UC-MSC) have shown promise in alleviating symptoms. However, which types of mesenchymal stem cells (MSCs) have the best therapeutic outcomes remain uncertain.

**Method:**

We systematically searched PubMed, OVID, Web of Science, and the Cochrane Library until January 1, 2024. The study evaluated five endpoints: Visual Analog Score (VAS) for Pain, Range of Motion (ROM), Whole-Organ Magnetic Resonance Imaging Score (WORMS), Western Ontario McMaster Universities Osteoarthritis Index (WOMAC), and adverse events (ADs). Standard meta-analysis and network meta-analysis were performed using Stata 16.0.

**Results:**

Fifteen studies involving 585 patients were included in the meta-analysis. Standard meta-analysis revealed significant improvements with MSCs in VAS score (*P* < 0.001), knee ROM (*P* < 0.001), and WOMAC (*P* < 0.016) compared to traditional therapy. In the network meta-analysis, autologous MSCs significantly improved VAS score [SMD = 2.94, 95% CI (1.90, 4.56)] and knee ROM [SMD = 0.26, 95% CI (0.08, 0.82)] compared to traditional therapy. Similarly, BM-MSC significantly improved VAS score [SMD = 0.31, 95% CI (0.11, 0.91)] and knee ROM [SMD = 0.26, 95% CI (0.08, 0.82)] compared to hyaluronic acid. However, compared with traditional therapy, autologous or allogeneic MSCs were associated with more adverse reactions [SMD = 0.11, 95% CI (0.02, 0.59)], [SMD = 0.13, 95% CI (0.002, 0.72)]. Based on the surface under the cumulative ranking results, autologous BM-MSC showed the most improvement in ROM and pain relief in KOA patients, UC-MSC (SUCRA 94.1%) were most effective for positive WORMS, and AD-MSC (SUCRA 70.6%) were most effective for WOMAC-positive patients.

**Conclusion:**

MSCs transplantation effectively treats KOA patients, with autologous BM-MSC potentially offering more excellent benefits.

**Supplementary Information:**

The online version contains supplementary material available at 10.1186/s13018-024-04846-1.

## Introduction

Osteoarthritis (OA) stands as a prominent cause of joint pain and disability among adults, with over 30 million symptomatic adults in the United States alone [1]. The estimated annual cost of OA and related disorders is $461 billion when considering direct and indirect expenses [2]. While OA can affect any joint, the knee is the most commonly affected, with 10% of men and 13% of women over 60 experiencing symptomatic knee osteoarthritis (KOA) [3]. This prevalence is expected to rise due to increasing life expectancy and the obesity epidemic [4, 5]. Presently, nonoperative treatment options include physical therapy, nonsteroidal anti-inflammatory drugs, and intraarticular injections of corticosteroids and hyaluronic acid (HA) [6, 7]. Cartilage degeneration remains irreversible despite these options, highlighting the need for novel and practical treatment approaches for KOA to address its complex pathology [8].

Recent extensive studies have suggested mesenchymal stem cells (MSCs) as a promising alternative for treating symptomatic KOA due to their multifaceted effects on the local environment [9–11]. MSCs have shown effectiveness in healing and regenerating cartilage defects, potentially enhancing cartilage regeneration and mitigating the degenerative process when injected intra-articularly in KOA cases [12]. MSCs possess diverse properties, including anti-inflammatory, anti-microbial, analgesic, regenerative, immunomodulatory, and immune-evasive capabilities [7]. These cells can be sourced from bone marrow, adipose tissue, umbilical cord, amniotic fluid, placenta, menstrual blood, dental pulp, and endometrium. Bone marrow, adipose tissue, and umbilical cord are the most accessible sources [13]. Nonetheless, debates persist among orthopedic and translational medicine researchers regarding the choice of MSC types and sources for KOA treatment.

Bone marrow-derived mesenchymal stem cells (BM-MSC) have been extensively studied and have shown potential to improve knee pain and function and restore cartilage morphology in some instances [12, 14]. Adipose tissue-derived mesenchymal stem cells (AD-MSC) are garnering attention due to their simplicity in extraction, low complication rates, and minimal donor site morbidity [15, 16]. Additionally, umbilical cord-derived mesenchymal stem cells (UC-MSC) exhibit promising clonogenic, proliferative, and migratory capabilities and enhanced secretion of chondrogenic factors [17].

Numerous meta-analyses have explored the efficacy of single MSCs in treating KOA and chondral defects, primarily focusing on pain and physical function [18–20]. While these studies support the use of MSCs in clinical practice, few systematic reviews have evaluated the relative efficacy and safety of different MSCs types and sources in KOA treatment or compared these strategies. Thus, we designed and conducted this network meta-analysis to comprehensively assess the clinical efficacy and safety of various MSC sources and types for treating KOA and identify the optimal strategy.

## Methods

The meta-analysis followed the requirements of the Cochrane Handbook for Systematic Reviews of Interventions [21], registered in PROSPERO (CRD42022351142), and PRISMA reporting guidelines [22].

### Data source and search strategy

Two independent reviewers independently searched the following four databases for comprehensive literature information: PubMed, OVID, Web of Science, and the Cochrane Library; searches were conducted from their inception until January 1, 2024, on all related papers. The following free words or phrases and their abbreviations were used to develop a search strategy. The literature search strategy consisted of MeSH terms and the free words “knee osteoarthritis” and “mesenchymal stem cells” (Supplementary materials. Table 1). In addition, reference sections in the searched articles were manually checked to ensure that no relevant studies were overlooked.

**Table 1:** Study Inclusion and Exclusion Criteria

Inclusion Criteria	Exclusion Criteria
(a) Patients diagnosed with KOA, regardless of race, sex, age, disease course, and severity were included in this study (b) The treatment group was treated with MSCs. Patients in the control group received traditional treatment but did not receive stem cells therapy (c) The type of study was randomized controlled trials (d) Articles in the English language (e) Articles published in peer-reviewed journals	(a) Studies reporting the outcomes after MSCs therapy to the knee combined with other types of stem cells therapy (b) Studies reporting the outcomes after multiple MSCs therapy (c) Observational studies and interventional studies without a comparator group (d) Review articles and in-vitro studies involving stem cells therapy (e) Animal studies involving stem cells therapy for knee osteoarthritis models (f) The data in the study is relevant but could not be extracted
KOA, knee osteoarthritis; MSCs, mesenchymal stem cells.	

### Study selection criteria

The meta-analysis included clinical studies investigating the outcomes of patients who received MSCs therapy in the knee joint to treat osteoarthritis of any degree. Each randomized controlled trials (RCTs) compared MSC transplantation directly with other established treatment modalities, highlighting the comparative effectiveness of these approaches. Studies examining patients receiving other cell therapies alone or in combination were excluded. Table 1 presents the study inclusion and exclusion criteria in detail.

### Types of studies

We included only RCTs that tested the effectiveness and safety of MSCs in treating KOA.

### Types of participants

This study included participants who were diagnosed with KOA. To ensure that all relevant studies were included, we included all patients with KOA regardless of their age, the cause, the type, the time, the site, or the degree of KOA, as long as they met the inclusion criteria outlined in Table 1.

### Types of interventions

The included studies investigated a variety of interventions. Specifically, the treatment groups received MSCs therapy, which was sometimes combined with other conventional treatments such as HA or platelet-rich plasma injection. The control groups received placebo or other conventional treatments. There were no specific limitations on the dose, frequency, or method of administration of MSCs. The detailed interventions for each study are provided in Tables 2 and 3.

**Table 2:** Characteristics of Included Studies

No	Study	County	Sample size(treatment/control)	Kellgren-Lawrence Grade	Mean Age (years)		Male/Female		Outcomes Measures	Follow-up(months)
					Treatment Group	Control Group	Treatment Group	Control Group		
1	Bastos(2019)	Brazil	16/17	I-IV	55.7 ± 7.8	55.9 ± 13.4	10/6	9/8	ROM	12
2	Emadedin(2018)	Iran	19/24	II-IV	51.7 ± 9.2	54.7 ± 5.3	12/7	15/9	WOMAC	6
3	Freitag(2019)	Australia	20/10	II-III	54.6 ± 6.3	51.5 ± 6.1	11/9	1/9	WOMAC/ADs	12
4	Garay-Mendoza(2017)	Mexico	30/31	-	55.57 ± 12.02	59.32 ± 1.85	7/23	9/22	WOMAC/VAS/ADs	6
5	Garza(2020)	USA	16/13	II-III	60.5 ± 7.95	57.1 ± 9.14	15/11	7/6	WOMAC/ADs	12
6	Goncars(2017)	Latvia	28/28	II-III	53.44 ± 15	58.55 ± 13	15/13	18/7	ADs	12
7	Gupta(2016)	India	40/20	II-III	58.1 ± 8.23	59.4 ± 8.27	12/28	4/16	WOMAC/VAS/WORMS/ADs	12
8	Koh(2014)	South Korea	25/25	IV	54.2 ± 9.3	54.4 ± 11.3	8/17	8/17	VAS	16
9	Kuah(2018)	Australia	16/4	II-IV	50.8 ± 7.29	55 ± 10.42	11/5	1/3	WOMAC/VAS/ADs	12
10	Lamo‑Espinosa(2018)	Spain	9/18	II-IV	60.6	65.9	7/2	10/6	WOMAC/VAS/ADs	48
11	Lamo‑Espinosa(2020)	Spain	26/24	II-IV	54.6	56	16/10	17/7	ROM/WOMAC/VAS/WORMS/ADs	12
12	Lee(2019)	South Korea	12/12	II-IV	62.2 ± 6.5	63.2 ± 4.2	3/9	3/9	ROM/WOMAC/VAS/ADs	6
13	Lu(2019)	China	27/26	I-III	55.53 ± 9.19	59.64 ± 5.97	3/24	3/23	WOMAC/VAS/ADs	12
14	Matas(2019)	Chile	10/9	II-III	54.8 ± 4.5	56.5 ± 4.1	5/5	4/5	WOMAC/VAS/WORMS/ADs	13
15	Vega(2015)	Spain	15/15	II-IV	56.6 ± 9.24	57.3 ± 9.09	6/9	5/10	WOMAC/VAS/ADs	12

VAS: Visual Analog Score for Pain; ROM: Range of motion; WORMS: Whole-Organ Magnetic Resonance Imaging Score; WOMAC: Western Ontario McMaster Universities Osteoarthritis Index; ADs: adverse events

**Table 3:** MSCs Transplantation Protocol of the Included Studies

Study	Harvest location	MSCs Type	MSCs Source	Transplantation times	MSCs No.	Intervention	Control	Outcomes Measures
Bastos(2019)	posterior iliac crests	BM-MSC	Auto	1	40 × 10^6^	BM-MSC	Corticosteroid injections	ROM
Emadedin(2018)	iliac crests	BM-MSC	Auto	1	40 × 10^6^	BM-MSC	saline	WOMAC
Freitag(2019)	abdomen	AD-MSC	Allo	1	100 × 10^6^	AD-MSC	HA	WOMAC/ADs
Garay-Mendoza(2017)	posterior iliac crests	BM-MSC	Auto	1	10 mL concentrate of BM-MSC	BM-MSC	oral acetaminophen	WOMAC/VAS/ADs
Garza(2020)	-	AD-MSC	Allo	-	-	AD-MSC	placebo injections	WOMAC/ADs
Goncars(2017)	iliac crest	BM-MSC	Auto	1	Indirectly mentioned (A total up of 45 ml of bone marrow)	BM-MSC	HA	ADs
Gupta(2016)	-	BM-MSC	Allo	1	25 × 10^6^	BM-MSC	HA	WOMAC/VAS/WORMS/ADs
Koh(2014)	buttocks	AD-MSC	Auto	1	4 × 10^6^	AD-MSC + HTO	PRP + HTO	VAS
Kuah(2018)	-	AD-MSC	Allo	4	6.7 × 10^6^	AD-MSC	placebo injections	WOMAC/VAS/ADs
Lamo‑Espinosa(2018)	iliac crest	BM-MSC	Auto	1	100 × 10^6^	BM-MSC + HA	HA	WOMAC/VAS/ADs
Lamo‑Espinosa(2020)	iliac crest	BM-MSC	Auto	3	100 × 10^6^	BM-MSC + PRP	PRP	ROM/WOMAC/VAS/WORMS/ADs
Lee(2019)	abdomen	AD-MSC	Auto	1	1 × 10^8^	AD-MSC	saline injections	ROM/WOMAC/VAS/ADs
Lu(2019)	abdomen	AD-MSC	Auto	4	5 × 10^7^	AD-MSC	HA	WOMAC/VAS/ADs
Matas(2019)	umbilical cords	UC-MSC	Allo	2	20 × 10^6^	UC-MSC	HA	WOMAC/VAS/WORMS/ADs
Vega(2015)	posterior iliac crests	BM-MSC	Allo	1	40 × 10^6^	BM-MSC	HA	WOMAC/VAS/ADs

PRP: platelet-rich plasma; HTO: high tibial osteotomy; HA: Hyaluronic acid; Auto: autologous mesenchymal stem cells, Allo: allogeneic mesenchymal stem cells; BM-MSC: bone marrow-derived mesenchymal stem cells; AD-MSC: adipose tissue-derived mesenchymal stem cells; UC-MSC: umbilical cord-derived mesenchymal stem cells; VAS: Visual Analog Score for Pain; ROM: Range of motion; WORMS: Whole-Organ Magnetic Resonance Imaging Score; WOMAC: Western Ontario McMaster Universities Osteoarthritis Index; ADs: adverse events

### Types of outcome measures

The outcome measures we evaluated included Visual Analog Score (VAS) for Pain [23], Range of motion (ROM), Whole-Organ Magnetic Resonance Imaging Score (WORMS) [20, 24], Western Ontario McMaster Universities Osteoarthritis Index (WOMAC) [25], and adverse events (ADs).

### Data extraction and management

Using a standardized form, data were independently extracted and checked by two independent reviewers (Liu and Li), and another reviewer (Yin) adjusted the differences of opinion. The form contains the following items: the first author’s name, journal, year of publication, country of study, language, sample size, sex, age, diagnosis method, Kellgren-Lawrence Grade, MSCs Harvest location, MSCs Type, MSCs Source, details of treatment and control intervention, treatment duration, intervention duration, follow-up, outcome measure, and summary of results according to the Cochrane Handbook for Systematic Reviews of Interventions (version 6.1.0) [26].

### Bias assessment of the included studies

Two reviewers (Liu and Li) independently assessed the risk of bias utilizing the risk of bias tool developed by Cochrane [27]. The following criteria were used to evaluate each trial: random sequence generation (selection bias), allocation concealment (selection bias), blinding of participants and personnel (performance bias), blinding of outcome assessment (detection bias), incomplete outcome data (attrition bias), selective outcome reporting (reporting bias), and other bias. The judging criteria were rated as low risk of bias, unclear of bias, or low risk of bias. When two reviewers could not reach an agreement, a third experienced reviewer (Yin) made the final decision.

### Quality of evidence

Based on the risk of bias tool developed by Cochrane, two independent reviewers (Liu and Li) assessed the methodological quality of the included studies. When disagreements could not be resolved through discussion, a third experienced reviewer (Yin) added his perspective and made the final decision. Each study was assessed for selection bias, performance bias, detection bias, attrition bias, reporting bias, and other biases. Each domain was rated as high risk of bias, low risk of bias, or unclear risk of bias.

### Data synthesis and statistical analysis

The methodological quality and risk of bias assessment for all included studies were conducted using RevMan (Review Manager, Version 5.4) software. Network meta-analyses (NMAs) and standard meta-analyses were performed using STATA 16.0 software (Stata Corporation, Texas) for dichotomous data such as ADs, odds ratios (OR), and their corresponding 95% confidence intervals (CI) were calculated. Continuous outcomes, including ROM, VAS, WOMAC, and WORMS, were presented as standard mean difference (SMD) with 95% CI. Heterogeneity among the trials was assessed using the chi-square test and I2 statistics. A fixed-effects model was utilized for data analysis when *p* > 0.1 and I2 < 50%; otherwise, a random-effects model was applied for *p* < 0.1 and I2 > 50%.

In network meta-analysis, estimates are derived from either indirect or mixed evidence. When direct evidence is lacking, the analysis estimates indirect evidence based on trials comparing interventions with a common comparator. Conversely, when direct evidence is available, a mixed treatment effect is estimated using the weighted average of both direct and indirect evidence [28]. Pairwise meta-analyses based on random effects models were employed to derive effects from direct evidence. Global inconsistency and node-split tests were conducted, and the consistency model was adopted if no inconsistency was detected (p-value of Z-test > 0.05)^29^.

Publication bias for each endpoint was assessed using funnel plots and Egger’s tests in Stata. Surface under the cumulative ranking (SUCRA), treatment rankings, and probabilities of the best treatment were determined using Bayesian frameworks based on random-effects models. The SUCRA score, ranging from 0 to 100, indicates the treatment’s relative efficacy, with higher scores representing better treatments. These rankings and probabilities aid in understanding the preferred order of treatments for the average patient, as determined by clinicians and policymakers. However, it is essential to note that treatment effects are most significant, as a favorable rank does not necessarily imply a substantial or clinically significant effect [30].

## Results

### Literature Selection

**Fig. 1:**
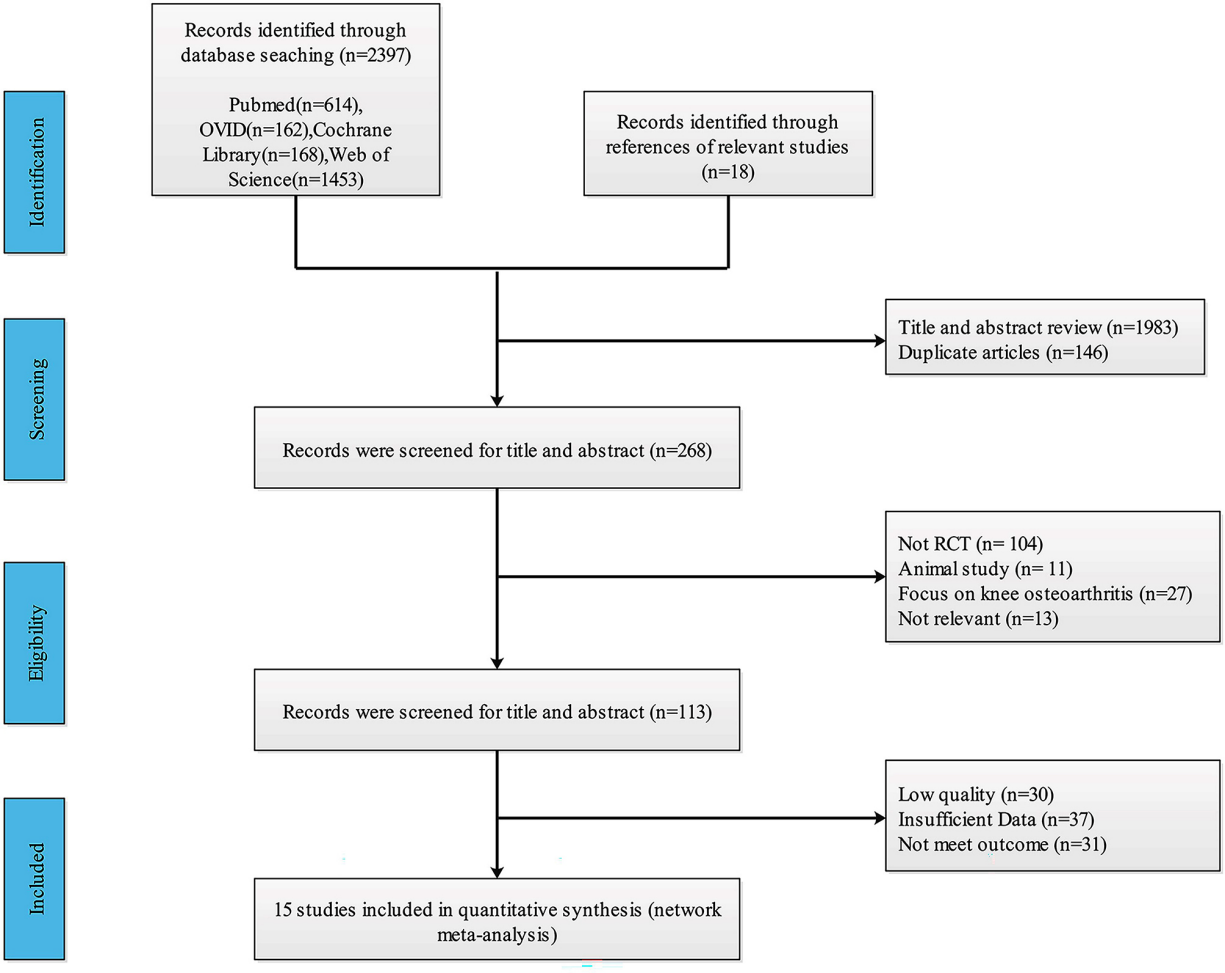
Flow chart of study search and selection process

A total of 2397 studies were initially identified through a thorough search of 4 electronic databases and 18 additional studies referenced in relevant literature. Subsequently, 1983 studies were excluded after screening their titles and abstracts against predefined inclusion criteria. Additionally, 146 duplicated studies were removed. This process left 268 trials for further consideration. After reviewing the full texts of these trials, an additional 131 studies were excluded based on the exclusion criteria. Ultimately, 15 studies involving 585 patients met the criteria for inclusion in the meta-analysis (Fig. 1).

### Characteristics of included trials

The characteristics of the included trials, along with intervention types, are summarized in Table 2. All included studies demonstrated no significant differences in baseline characteristics. These randomized controlled trials (RCTs) involved 585 patients with Kellgren-Lawrence Grade ranging from I to IV. Among them, 294 patients received intra-articular injections of MSCs, while 261 patients were treated with traditional drugs, primarily HA. The follow-up duration varied from 6 to 48 months, most falling within the 6 to 12 months range.

Of the fifteen studies, nine utilized autologous MSCs, while the remainder employed allogeneic MSCs. Six out of fifteen studies utilized MSCs derived from adipose tissue, eight from bone marrow, and one from umbilical cords. In all studies, cell transplantation only involved intra-articular injection in the treatment groups. The dosage of transplanted MSCs varied among the studies, and the frequency of transplantation ranged from one to four times, with most studies employing a single treatment session. Table 3 provides an overview of the general characteristics of the MSCs transplantation protocols used in the included studies. Clinical outcomes assessed included ROM, VAS score, WOMAC, WORMS, and ADs.

### Methodological quality of included trials

**Fig. 2:**
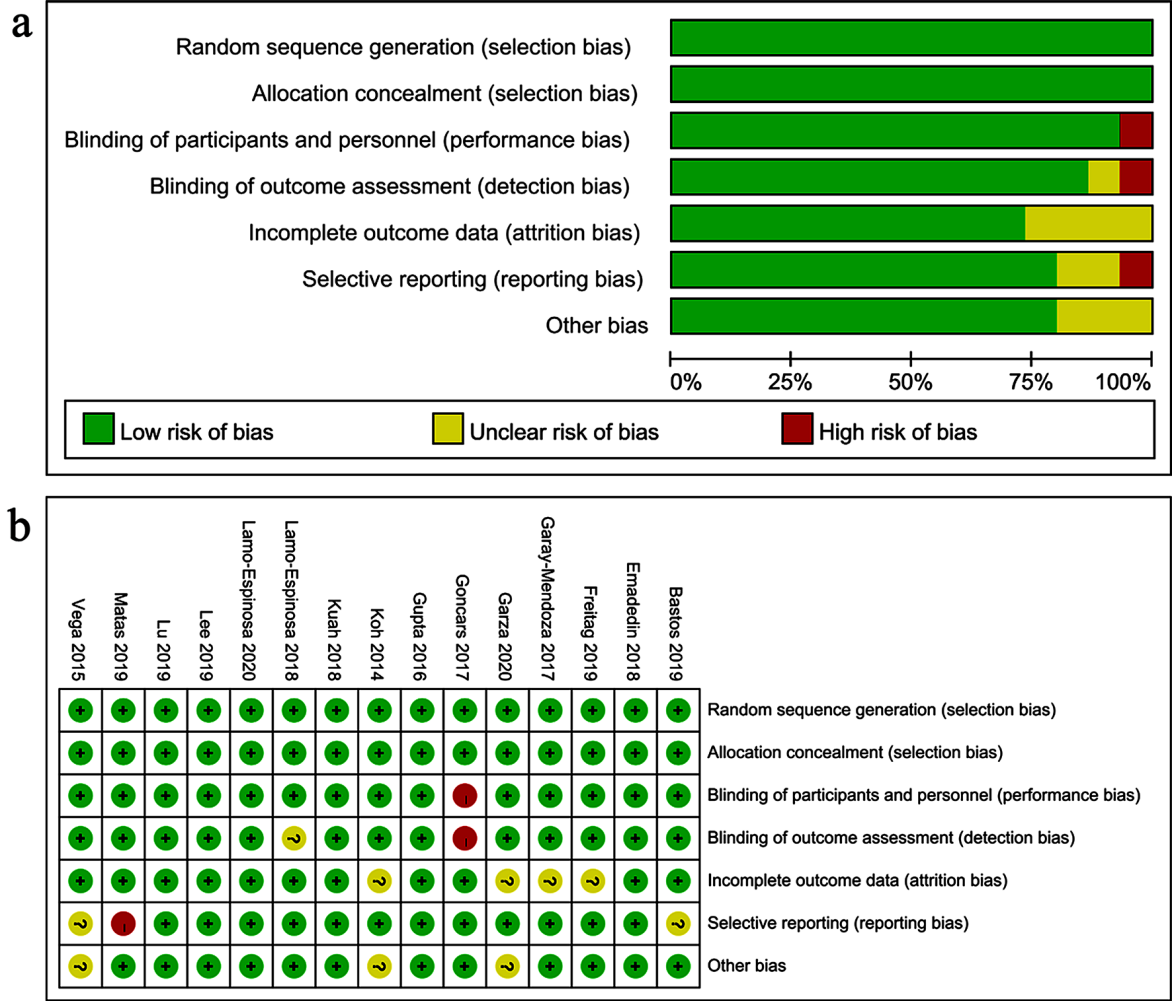
Risk of bias. **a** Risk of bias graph. **b** Risk of bias summary

To assess the risk of bias in the studies included, we used the standard Cochrane collaborative tool, and the risk of bias assessment for the included studies is shown in Fig. 2. Overall, the study included in this review was of acceptable methodological quality.

### Standard meta-analysis

#### VAS score for pain

**Fig. 3:**
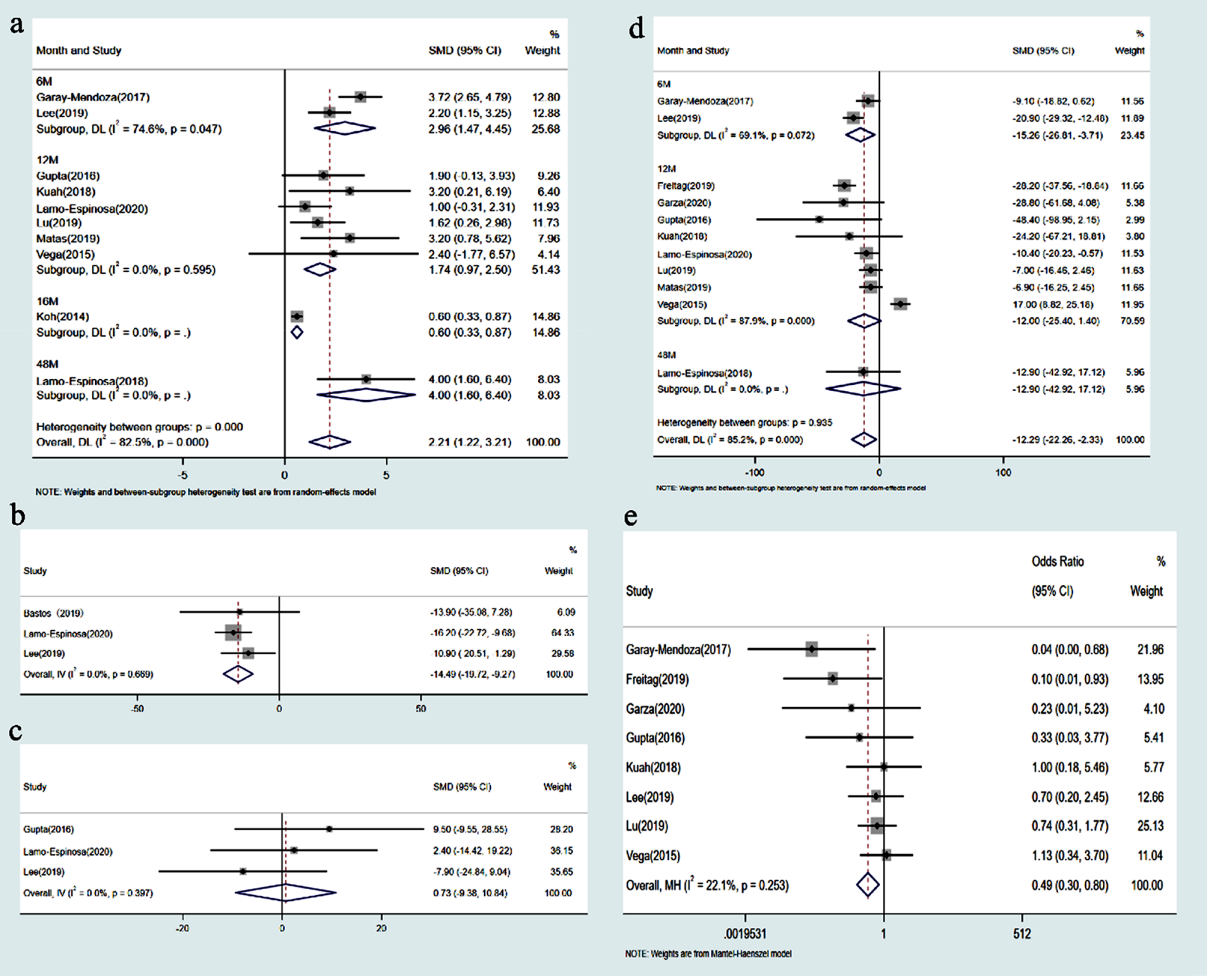
Forest plot of comparison: MSC transplantation group versus control group. **a** VAS score; **b** knee ROM; **c** WORMS score; **d** WOMAC; **e** Adverse events

Ten studies, comprising 319 cases, reported VAS scores. Analysis revealed high statistical heterogeneity among the studies (p for heterogeneity < 0.0001, I2 = 82.5%). Subgroup analysis based on follow-up duration (6, 12, 16, and 48 months) was conducted, with one subgroup maintaining an I2 of over 50%, necessitating a random-effects model. The meta-analysis indicated a significant reduction in VAS scores with MSCs transplantation therapy compared to the control group (SMD = 2.21; 95% CI = [1.22, 3.21]; *P* < 0.001). (Fig. 3a)

#### ROM

Three studies involving 121 patients reported knee ROM. With an I2 of 0.0% and p for heterogeneity = 0.669, indicating low heterogeneity, a fixed-effect model was applied for meta-analysis. Results showed a significant improvement in knee ROM with MSCs transplantation therapy compared to the control group (SMD = -14.49; 95% CI = [-19.72, -9.27]; *P* < 0.001). (Fig. 3b)

#### WORMS

WORMS were reported in three studies comprising 100 patients. Low heterogeneity was observed among the included studies (I2 = 0.0%, p for heterogeneity = 0.397), warranting a fixed-effect model. However, no significant difference in WORMS was noted between the MSCs transplantation therapy group and the control group (SMD = 0.73; 95% CI = [-9.38, 10.84]; *P* = 0.888). (Fig. 3c)

#### WOMAC

Eleven studies, encompassing 301 cases, reported WOMAC total scores. Analysis revealed a high level of statistical heterogeneity among the studies (p for heterogeneity < 0.0001, I2 = 85.2%). Subgroup analysis based on follow-up duration (6, 12, and 48 months) was conducted, with two out of three subgroups showing an I2 more significant than 50%, leading to the utilization of a random-effects model. The results indicated a higher WOMAC total score in the MSCs transplantation therapy group compared to the control group (SMD = -12.29; 95% CI = [-22.26, -2.33]; *P* < 0.016). (Fig. 3d)

#### ADs

Eight studies involving 254 patients described adverse events during treatment and follow-up. None reported severe complications with permanent effects such as tumors, abnormal tissue proliferation, or immune reactions. The most commonly reported adverse reactions included minor discomfort, bruising, fever, and headache, which resolved spontaneously or with symptomatic treatment. Meta-analysis showed low heterogeneity among the included studies (I2 = 22.1%, *P* = 0.235), warranting a fixed-effects model for analysis. MSCs transplantation therapy was associated with a higher incidence of adverse events compared to the control group (OR = 0.49; 95% CI = [0.30, 0.80]; *P* = 0.005) (Fig. 3e). The remaining studies did not report any adverse events or side effects.

### Network meta-analysis

#### Inconsistency analysis

We calculated the absolute difference between direct and indirect evidence using the relative odds ratio (ROR) with 95% confidence intervals. Consistency between direct and indirect evidence is indicated when ROR approximates one, or the 95% CI includes 0. No closed loop was formed in analyzing VAS scores, WORMS, WOMAC, and ADs outcome measures. Hence, no inconsistency analysis was performed. Additionally, no significant inconsistency was detected for ROM comparisons across different sources and types of MSCs, suggesting that the consistency model is more appropriate than the inconsistency model. Consequently, the consistency model was applied for VAS scores, ROM, WORMS, WOMAC, and ADs.

#### Comparison between different MSCs sources

##### Network plot

**Fig. 4:**
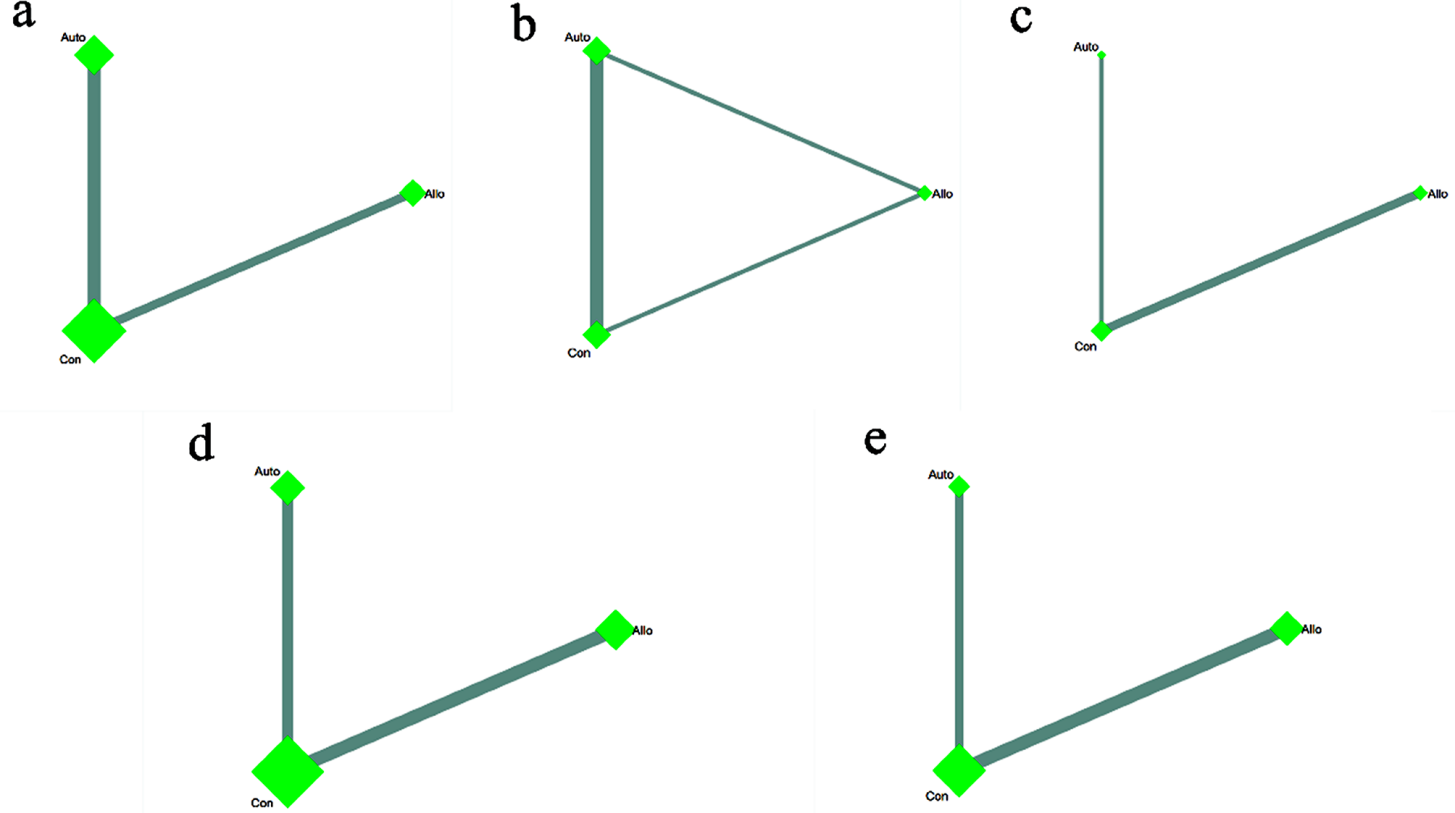
Network meta-analysis of different MSCs sources, Network plot of the subgroup: **a** VAS score; **b** knee ROM; **c** WORMS score; **d** WOMAC; **e** Adverse events. Auto: autologous mesenchymal stem cells, Allo: allogeneic mesenchymal stem cells, Con: control group

We generated five network plots for the five outcomes, each representing different sources of MSCs. A summary network plot of these comparisons is presented in Fig. 4a–e.

##### VAS score for pain

**Fig. 5:**
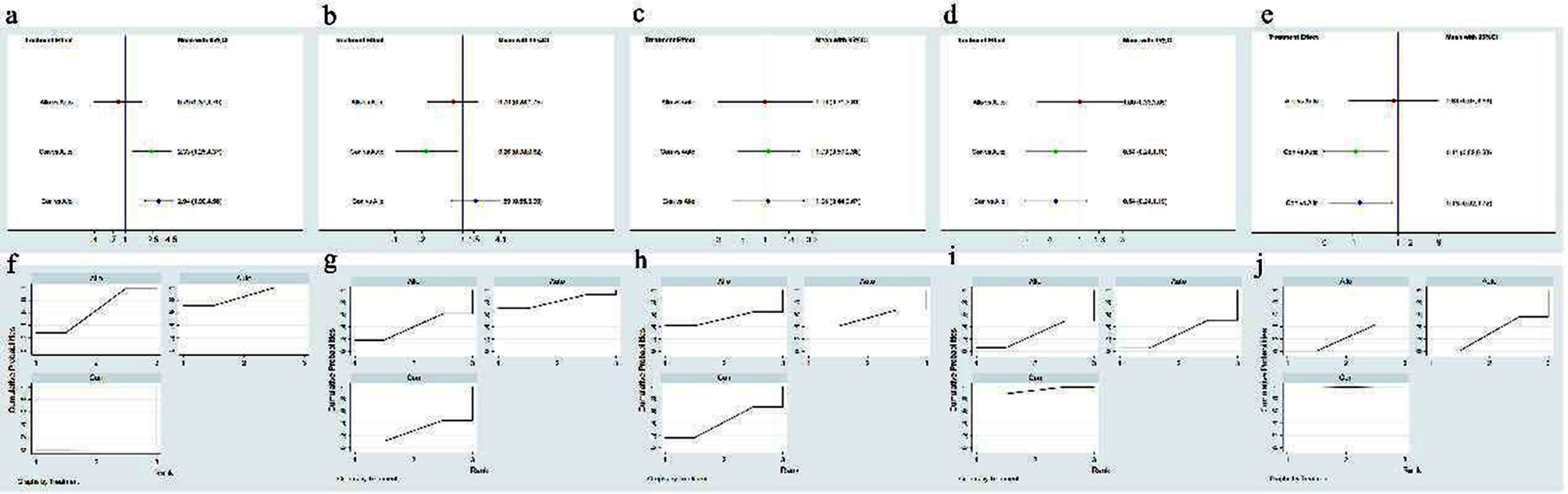
Network meta-analysis of different MSCs sources. **a-e** Forest plot represents the direct and indirect comparison; **f-j** the surface under the cumulative ranking curves for different outcomes. From left to right are VAS score, knee ROM, WORMS score, WOMAC, and Adverse events, respectively

Ten pairwise comparisons were analyzed in this network. NMAs revealed a significant reduction in VAS scores with both autologous MSCs therapy [SMD = 2.33, 95% CI (1.25, 4.37)] and allogeneic MSCs therapy [SMD = 2.94, 95% CI (1.90, 4.56)] compared to traditional therapy. However, no significant differences were observed between autologous and allogeneic MSCs therapy (Fig. 5a). Autologous MSCs therapy demonstrated the lowest VAS scores (SUCRA 86.1%), followed by allogeneic MSCs therapy (SUCRA 63.8%). (Fig. 5f)

##### ROM

Three studies were assessed in this network, one of which was a 3-arm study. NMAs showed that autologous MSCs therapy [SMD = 0.26, 95% CI (0.08, 0.82)] improved knee ROM compared to traditional therapy. However, no significant differences were found between allogeneic MSCs therapy and traditional therapy, nor between autologous and allogeneic MSCs therapy (Fig. 5b). Autologous MSCs therapy had the highest probability of being the best option for improving knee ROM (SUCRA 81.7%), followed by allogeneic MSCs therapy (SUCRA 40.2%) and traditional therapy (SUCRA 28.1%). (Fig. 5g)

##### WORMS

Three articles assessing knee WORMS were included in this network. Similar to standard meta-analysis results, no significant differences in WORMS scores were observed in the network meta-analysis. According to the SUCRA rank, autologous MSCs therapy was theoretically the best strategy for positive WORMS scores (SUCRA 54.6%), followed by allogeneic MSCs therapy (SUCRA 53.3%), with traditional therapy having the lowest outcome (SUCRA 42.1%). (Figure 5c and h)

##### WOMAC

Eleven articles were included in the WOMAC network. Similar to the WORMS network results, no significant differences were found in WOMAC outcomes through a network meta-analysis. According to the SUCRA rank, traditional therapy was the most effective treatment strategy for positive WOMAC scores (SUCRA 94.1%), followed by autologous MSCs therapy (SUCRA 28.2%) and allogeneic MSCs therapy (SUCRA 27.7%). (Figure 5d and i)

##### ADs

Eight studies assessed adverse reactions in this network. Both autologous MSCs therapy [SMD = 0.11, 95% CI (0.02, 0.59)] and allogeneic MSCs therapy [SMD = 0.13, 95% CI (0.002, 0.72)] were associated with increased adverse events compared to traditional therapy. No significant difference was observed between autologous and allogeneic MSCs therapy. According to SUCRA values, traditional therapy was the best option to avoid adverse events (SUCRA 99.1%), followed by autologous MSCs therapy (SUCRA 28.9%) and allogeneic MSCs therapy (SUCRA 22.0%), which might be the least preferable strategy. (Figure 5e and j)

#### Comparison between different MSCs types

##### Network plot

**Fig. 6:**
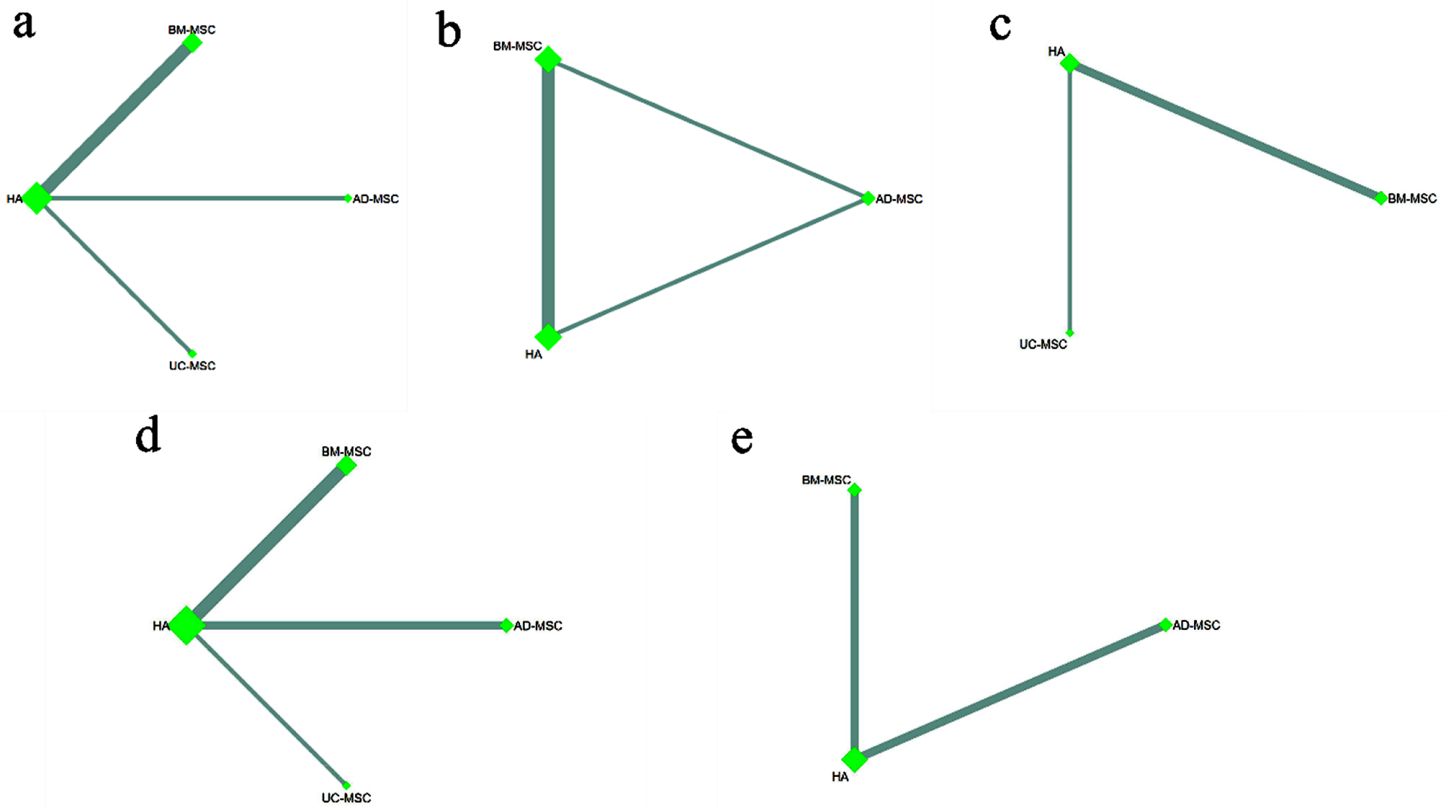
Network meta-analysis of different MSCs types, Network plot of the subgroup: **a** VAS score; **b** knee ROM; **c** WORMS score; **d** WOMAC; **e** Adverse events. BM-MSC: bone marrow-derived mesenchymal stem cells; AD-MSC: adipose tissue-derived mesenchymal stem cells; UC-MSC: umbilical cord-derived mesenchymal stem cells, HA: Hyaluronic acid

We generated five network plots for each outcome, each representing different types of MSCs. Figure 6a–e presents a summary network plot of these comparisons.

##### VAS scores for pain

**Fig. 7:**
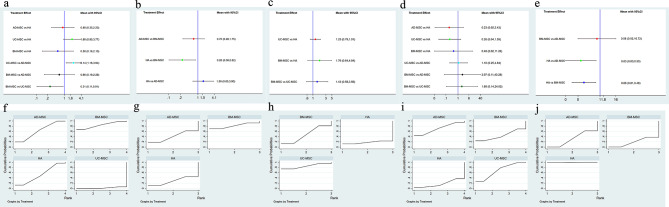
Network meta-analysis of different MSCs types. **a-e** Forest plot represents the direct and indirect comparison; **f-j** the surface under the cumulative ranking curves for different outcomes. From left to right are VAS score, knee ROM, WORMS score, WOMAC, and Adverse events, respectively

Five pairwise comparisons were analyzed in this network. NMAs indicated a significant decrease in VAS scores with AD-MSC therapy [SMD = 2.14, 95% CI (1.16, 3.94)] and BM-MSC therapy [SMD = 0.31, 95% CI (0.11, 0.91)] compared to UC-MSC therapy. According to the SUCRA rank, BM-MSC therapy was the most effective strategy (SUCRA 83.9%), followed by AD-MSC therapy (SUCRA 62.1%), HA therapy (SUCRA 52.0%), and UC-MSC therapy (SUCRA 1.9%) being the least preferable option. (Figure 7a and f)

##### ROM

Three studies were assessed in this network, including one 3-arm study. NMAs showed that BM-MSC therapy [SMD = 0.26, 95% CI (0.08, 0.82)] improved knee ROM compared to HA therapy. However, no significant differences were observed between AD-MSC therapy and HA therapy, nor between AD-MSC therapy and BM-MSC therapy (Fig. 7b). In terms of SUCRA ranking probability, BM-MSC therapy may be the best option to improve knee ROM (SUCRA 78.0%), followed by HA therapy (SUCRA 41.3%) and AD-MSC therapy (SUCRA 30.7%). (Fig. 7g)

##### WORMS

Three articles for WORMS scores were included in this network. No significant differences were found in WORMS scores through network meta-analysis. According to the SUCRA rank, UC-MSC therapy (SUCRA 94.1%) was the most effective treatment strategy for positive WORMS scores, followed by BM-MSC therapy (SUCRA 28.2%) and HA therapy (SUCRA 27.7%). (Figure 7c and h)

##### WOMAC

The WOMAC network meta-analysis included six articles. Similar to the WORMS network results, no significant differences were observed in WOMAC outcomes through network meta-analysis. Based on SUCRA rank, AD-MSC therapy (SUCRA 70.6%) may be the most effective option for WOMAC-positive patients, followed by UC-MSC therapy (SUCRA 68.3%) and BM-MSC therapy (SUCRA 43.9%). HA therapy (SUCRA 17.2%) may be the least effective treatment option. (Figure 7d and i)

##### ADs

Four studies assessed adverse reactions in this network. Compared with HA therapy, AD-MSC therapy [SMD = 0.03, 95% CI (0.00, 0.50)] and BM-MSC therapy [SMD = 0.06, 95% CI (0.01, 0.48)] resulted in increased adverse reactions. No significant differences were found between BM-MSC therapy and AD-MSC therapy. SUCRA values indicated that HA therapy (SUCRA 99.6%) was the most effective strategy, followed by AD-MSC therapy (SUCRA 31.3%) and BM-MSC therapy (SUCRA 19.1%). (Figure 7e and j)

##### Publication bias

**Fig. 8:**
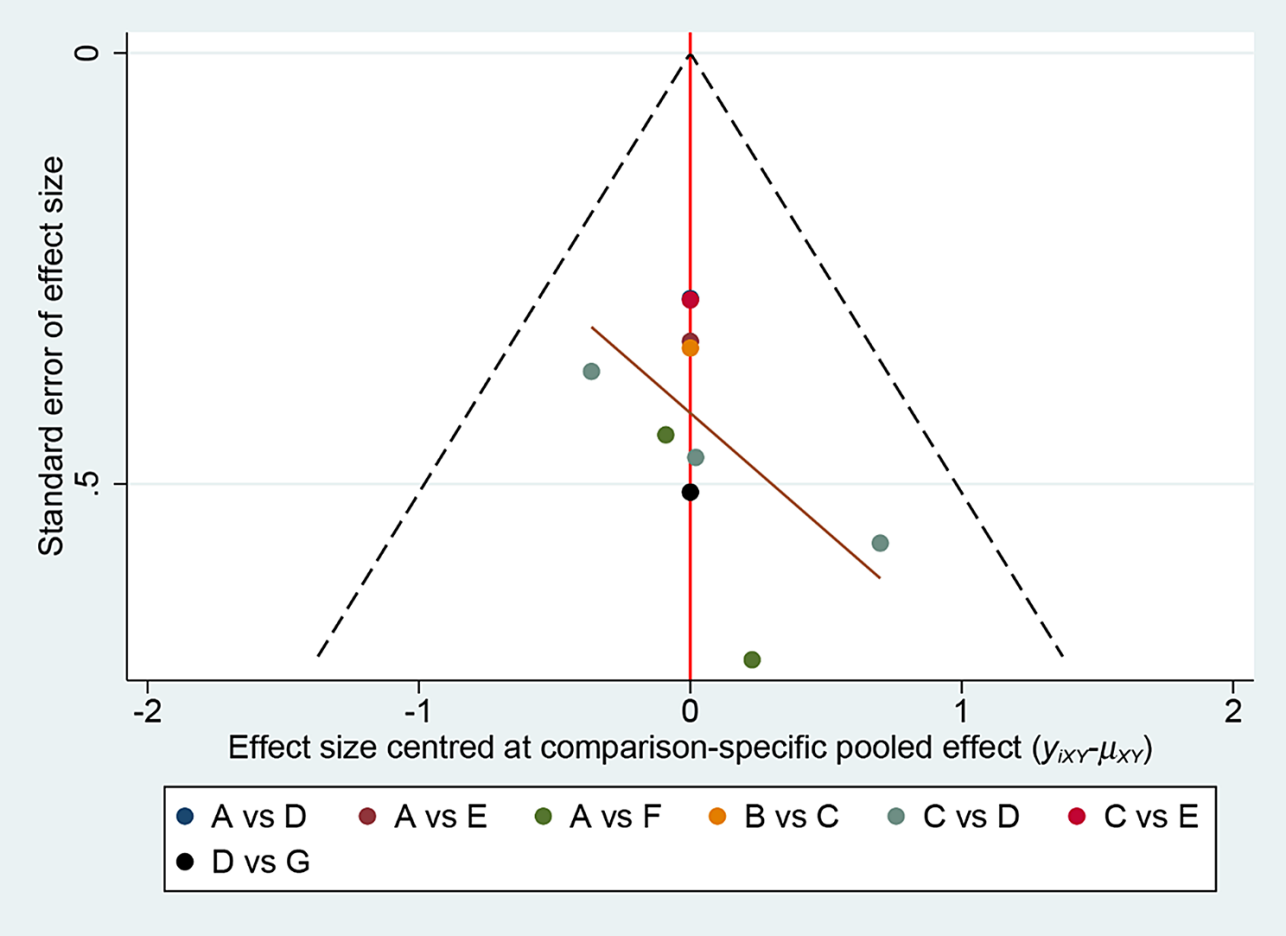
VAS as a marker of publication bias analysis

Publication bias was assessed using the funnel plot and Egger’s test. Although the funnel plot showed some asymmetry, possibly due to small sample sizes or publication bias (Fig. 8), Egger’s test did not reveal significant evidence of publication bias (*P* = 0.061), suggesting minimal publication bias. (See Supplementary materials, Table 2)

## Discussion

This trial assessed the safety and effectiveness of various sources and types of MSCs transplantation in patients with symptomatic KOA. Our study provides empirical evidence that MSCs transplantation effectively treats KOA patients. Specifically, our findings highlight that (1) autologous BM-MSC showed the most improvement in function and pain relief in KOA patients; (2) UC-MSC were most effective for positive WORMS, and AD-MSC were most effective for WOMAC-positive patients; (3) compared with traditional therapy, autologous or allogeneic MSCs were associated with more adverse reactions. To our knowledge, this is the first investigation to determine the optimal MSCs strategy for KOA and the initial comparison among BM-MSC, AD-MSC, and UC-MSC.

NMAs, by amalgamating direct and indirect comparisons, can augment study size, thereby enhancing statistical power [24]. According to NMAs findings, autologous MSC transplantation proved more effective than traditional therapy in terms of VAS scores and ROM. However, both autologous and allogeneic MSCs transplantations were associated with more reversible adverse reactions compared to traditional therapy, consistent with standard meta-analysis results. Considering clinical efficacy and safety probabilities, autologous MSCs transplants were more likely to be effective than allogeneic transplants. Moreover, BM-MSC showed the highest probability of being the most effective option in VAS scores and ROM. However, BM-MSC also elicited the highest frequency of adverse events, compared to HA with minimal complications, followed by AD-MSC. Among other MSC types, UC-MSC exhibited the highest probability of being the best treatment strategy for positive WORMS results. At the same time, AD-MSC ranked highest for WOMAC-positive patients. Except for BM-MSC and AD-MSC, which significantly reduced pain compared to UC-MSC, no significant differences were observed among the MSCs regarding ROM, WORMS, WOMAC, and adverse events.

The findings of previous meta-analyses align closely with those of our study concerning the effectiveness and safety of MSCs in treating KOA [18–20]. However, prior studies needed more clarity regarding the distinction between allogeneic and autologous MSCs and the impacts of various MSCs types. In contrast, our study offered the advantage of precisely defining the sources and types of MSCs. Additionally, we employed NMAs to rank subgroups derived from different cell sources that could not be directly compared. This approach allowed us to explore the optimal cell type through indirect comparisons, enhancing our understanding of the therapeutic efficacy of MSCs in KOA treatment.

Various MSCs sources can be harvested, including autologous bone marrow, adipose tissue, and allogeneic umbilical cord tissue. These cells exhibit differentiation, plasticity, immunomodulatory, immune evasive, and anti-inflammatory properties [23, 31, 32]. While MSCs have long been considered low immunogenic or immune-privileged, recent studies have indicated the production of antibodies and immune rejection against allogeneic MSCs, challenging this notion [33–35]. This suggests MSCs may not possess immune-privileged status as previously believed [35, 36]. Although the rejection of MSCs does not impact the efficacy of allogeneic MSCs therapy, safeguarding MSCs from immune responses and prolonging their persistence in vivo could enhance clinical outcomes and mitigate the development of antigen sensitivity [37, 38]. Our NMAs demonstrate that autologous MSCs outperform allogeneic MSCs regarding efficacy and safety. Thus, autologous MSCs may represent the most suitable cell source for treating KOA. However, this conclusion is drawn from indirect comparisons. Further well-designed and high-quality clinical randomized controlled trials are warranted to elucidate the impact of transplanted cell volume, frequency, duration, and KOA stage on treatment outcomes.

To achieve optimal functional outcomes in KOA treatment, selecting the appropriate MSCs source is crucial. Emerging evidence indicates that stem cell-based products such as BM-MSC, AD-MSC, and UC-MSC may ameliorate symptoms in osteoarthritis patients [7, 39]. Preclinical investigations evaluating clinical outcomes and cartilage repair post stem cell therapy in KOA have predominantly utilized BM-MSC, followed by AD-MSC and UC-MSC [25, 40]. Studies suggest that BM-MSC yield, particularly in the elderly, is relatively low compared to AD-MSC, despite the challenges associated with AD-MSC preparation compared to BM-MSC [41]. Notably, adipose tissue yields an MSC volume approximately 500 times greater than bone marrow [42, 43]. While some researchers assert that BM-MSC exhibits superior cartilage generation capabilities compared to AD-MSC, others propose that augmenting the stromal vascular fraction derived from AD-MSC with growth factors and cytokines can also enhance cartilage growth [44, 45]. Synovium-derived MSCs have been explored for assessing efficacy and functional outcomes in osteoarthritic knees, with several studies suggesting their superior chondrogenesis potential [46, 47]. Contrary to our findings, Lee et al. [48] observed that allogeneic human UC-MSC was more effective than autologous BM-MSC in cartilage regeneration in KOA, although clinical outcomes improved regardless of treatment type. Given the relatively recent exploration of UC-MSC in KOA treatment and the limited number of studies, further high-quality randomized controlled trials are warranted to validate their efficacy [49]. Regenerative and translational medicine holds promise in managing MSCs in KOA [50]. However, large RCTs are imperative to refine therapeutic protocols concerning MSCs type, isolation methods, and the quality and quantity of transplanted MSCs [19]. Addressing ethical concerns regarding tissue and cellular product manipulation and their functional outcomes is also essential [3, 13]. Given the complexities involved, an interdisciplinary approach is necessary to translate stem cell research into optimal clinical practice for KOA management.

This study has several limitations. Firstly, heterogeneity was observed across studies in most outcomes, possibly due to variations in treatment protocols, including the different traditional therapies utilized in the control groups of the included RCTs. This heterogeneity potentially impacts the validity of our findings. Secondly, the novelty of stem cell therapy for KOA and its limited availability in clinical practice restricted the number of patients and studies included in the analysis. Thirdly, studies were scarce assessing ROM and WORMS outcomes, particularly in AD-MSC and UC-MSC subgroups. Lastly, the included studies encompassed patients at different stages of KOA, contributing to the heterogeneity of the results. Therefore, further confirmation of our findings necessitates large multicentric trials with standardized dosage and frequency protocols and uniform outcome assessment measures, excluding adjuvant procedures.

## Conclusion

Transplanting MSCs in KOA yields superior outcomes to traditional therapies, notably enhancing function and alleviating pain. Furthermore, no significant disparities were noted when comparing various stem cell types and sources. However, BM-MSC therapy was most effective in improving the VAS and the ROM, while other types of MSCs were more effective in improving functional outcomes, such as WORMS and WOMAC scores. HA is the most advisable choice for treatment-associated adverse events, followed by AD-MSC and BM-MSCs. Although these adverse events are generally mild, they could adversely impact treatment compliance and satisfaction.

### Electronic supplementary material

Below is the link to the electronic supplementary material.

Supplementary Material 1

## Data Availability

No datasets were generated or analysed during the current study.
