# Supplementary material for: Transplantation of three mesenchymal stem cells for knee osteoarthritis, which cell and type are more beneficial? a systematic review and network meta-analysis
Source: J Orthop Surg Res. 2024 Jun 20;19:366. doi: 10.1186/s13018-024-04846-1 (PMC11188250; doi:10.1186/s13018-024-04846-1)
Supplement: Supplementary file 1 — Supplementary Material 1 [file 13018_2024_4846_MOESM1_ESM.doc]

### supplementary materials

**Supplementary Table 1. Search strategy.**

| Data source | Search terms | Total |
| --- | --- | --- |
| Pubmed | ((((Osteoarthritis, Knee[MeSH Terms]) OR (Knee Osteoarthritides[Title/Abstract])) OR (Knee Osteoarthritis[Title/Abstract])) OR (Osteoarthritis of Knee[Title/Abstract])) OR (Osteoarthritis of the Knee[Title/Abstract]) AND ((((((((((((((((((((((((((((((((((((((((((Mesenchymal Stem Cells[MeSH Terms]) OR (Stem Cell, Mesenchymal[Title/Abstract])) OR (Mesenchymal Stem Cell[Title/Abstract])) OR (Stem Cells, Mesenchymal[Title/Abstract])) OR (Bone Marrow Mesenchymal Stem Cells[Title/Abstract])) OR (Bone Marrow Mesenchymal Stem Cell[Title/Abstract])) OR (Bone Marrow Stromal Cells[Title/Abstract])) OR (Bone Marrow Stromal Cell[Title/Abstract])) OR (Bone Marrow Stromal Cells, Multipotent[Title/Abstract])) OR (Multipotent Bone Marrow Stromal Cell[Title/Abstract])) OR (Multipotent Bone Marrow Stromal Cells[Title/Abstract])) OR (Adipose-Derived Mesenchymal Stem Cells[Title/Abstract])) OR (Adipose Derived Mesenchymal Stem Cells[Title/Abstract])) OR (Adipose-Derived Mesenchymal Stromal Cells[Title/Abstract])) OR (Adipose Derived Mesenchymal Stromal Cells[Title/Abstract])) OR (Mesenchymal Stem Cells, Adipose-Derived[Title/Abstract])) OR (Mesenchymal Stem Cells, Adipose Derived[Title/Abstract])) OR (Adipose-Derived Mesenchymal Stem Cell[Title/Abstract])) OR (Adipose Derived Mesenchymal Stem Cell[Title/Abstract])) OR (Adipose Tissue-Derived Mesenchymal Stem Cell[Title/Abstract])) OR (Adipose Tissue Derived Mesenchymal Stem Cell[Title/Abstract])) OR (Adipose Tissue-Derived Mesenchymal Stem Cells[Title/Abstract])) OR (Adipose Tissue Derived Mesenchymal Stem Cells[Title/Abstract])) OR (Adipose Tissue-Derived Mesenchymal Stromal Cells[Title/Abstract])) OR (Adipose Tissue Derived Mesenchymal Stromal Cells[Title/Abstract])) OR (Adipose Tissue-Derived Mesenchymal Stromal Cell[Title/Abstract])) OR (Adipose Tissue Derived Mesenchymal Stromal Cell[Title/Abstract])) OR (Mesenchymal Stromal Cells[Title/Abstract])) OR (Mesenchymal Stromal Cell[Title/Abstract])) OR (Stromal Cell, Mesenchymal[Title/Abstract])) OR (Stromal Cells, Mesenchymal[Title/Abstract])) OR (Multipotent Mesenchymal Stromal Cells[Title/Abstract])) OR (Multipotent Mesenchymal Stromal Cell[Title/Abstract])) OR (Mesenchymal Stromal Cells, Multipotent[Title/Abstract])) OR (Mesenchymal Progenitor Cell[Title/Abstract])) OR (Mesenchymal Progenitor Cells[Title/Abstract])) OR (Progenitor Cell, Mesenchymal[Title/Abstract])) OR (Progenitor Cells, Mesenchymal[Title/Abstract])) OR (Wharton Jelly Cells[Title/Abstract])) OR (Wharton's Jelly Cells[Title/Abstract])) OR (Wharton's Jelly Cell[Title/Abstract])) OR (Whartons Jelly Cells[Title/Abstract])) OR (Bone Marrow Stromal Stem Cells[Title/Abstract]) | 614 |
| Cochrane | Knee Osteoarthritis and Mesenchymal Stem Cells | 168 |
| Web of science | **TOPIC**: (Knee Osteoarthritis and Mesenchymal Stem Cells)  Databases= WOS, KJD, MEDLINE, RSCI, SCIELO Timespan=All years  Search language=Auto | 1453 |
| OVID | (Knee Osteoarthritis and Mesenchymal Stem Cells).mp. [mp=title, abstract, original title, name of substance word, subject heading word, floating sub-heading word, keyword heading word, organism supplementary concept word, protocol supplementary concept word, rare disease supplementary concept word, unique identifier, synonyms] | 162 |
| Total | up to January 01, 2024 | 2482 |

**Supplementary Table 2**. **Egger's test**

| **Std. Eff** | **Coef.** | **Std. Err.** | ***t*** | ***P*** | **[95% Conf. Interval]** |
| --- | --- | --- | --- | --- | --- |
| slope | 0.359 | 0.260 | 1.38 | 0.206 | [-0.241, 0.959] |
| bias | 2.258 | 0.670 | 3.37 | 0.061 | [.7121, 3.804] |
| Test of H0: no small-study effects | | | | | |
